# Tailoring the Hybrid Magnetron Sputtering Process (HiPIMS and dcMS) to Manufacture Ceramic Multilayers: Powering Conditions, Target Materials, and Base Layers

**DOI:** 10.3390/nano12142465

**Published:** 2022-07-18

**Authors:** Bruno César Noronha Marques de Castilho, Felipe de Sousa Mazuco, Alisson Mendes Rodrigues, Pedro Renato Tavares Avila, Raira Chefer Apolinario, Philipp Daum, Fabiana Pereira da Costa, Romualdo Rodrigues Menezes, Gelmires de Araújo Neves, Christian Greiner, Haroldo Cavalcanti Pinto

**Affiliations:** 1 São Carlos School of Engineering—EESC, University of São Paulo—USP, São Carlos 13563-120, Brazil; brcn1000@gmail.com (B.C.N.M.d.C.); pedrorenatoavila@gmail.com (P.R.T.A.); raira.apolinario@usp.br (R.C.A.); 2 MAHLE Metal Leve S.A., Rodovia Anhanguera, Sentido Interior—Capital, km 49,7, Jundiai 13210-877, Brazil; felipe.mazuco@mahle.com; 3 Laboratory of Materials Technology (LTM), Department of Materials Engineering, Federal University of Campina Grande (UFCG), Campina Grande 58429-900, Brazil; alisson.mendes@professor.ufcg.edu.br (A.M.R.); fabiana.costa@estudante.ufcg.edu.br (F.P.d.C.); romualdo.menezes@ufcg.edu.br (R.R.M.); gelmires.neves@ufcg.edu.br (G.d.A.N.); 4 IAM-ZM MicroTribology Center µTC, Strasse am Forum 5, 76131 Karlsruhe, Germany; philipp.daum@iwm.fraunhofer.de (P.D.); christian.greiner@kit.edu (C.G.); 5 Institute for Applied Materials (IAM), Karlsruhe Institute of Technology (KIT), Kaiserstrasse 12, 76131 Karlsruhe, Germany

**Keywords:** ceramic multilayers, hybrid magnetron sputtering, HiPIMS, dcMS, mechanical properties, wear

## Abstract

The mechanical and wear behavior of CrN/CrAlN multilayers were improved by tailoring the experimental conditions of a hybrid magnetron sputtering process based on a high-power impulse (HiPIMS) and two direct current magnetron sputtering (dcMS) power supplies. To this end, the influence of the base layer and of the combination of Cr and CrAl targets, which were switched to the dcMS and HiPIMS power supplies in different configurations, were investigated with respect to the growth of ceramic CrN/CrAlN multilayers onto commercial gas-nitrided diesel piston rings. The microstructure, grain morphology, and mechanical properties were evaluated by field emission scanning electron microscopy (FE-SEM), atomic force microscopy (AFM), X-ray diffraction (XRD), and instrumented nanoindentation. Bench wear tests simulating the operation of a combustion engine were conducted against a gray cast iron cylinder liner under reciprocating conditions using 0W20 oil as a lubricating agent enriched with Al_2_O_3_ particles. The results revealed a significant increase in hardness, resistance to plastic strain, and wear resistance when two CrAl targets were switched to a HiPIMS and a dcMS power supply, and a Cr target was powered by another dcMS power supply. The compressive coating stresses were slightly reduced due to the soft Cr base layer that enabled stress relief within the multilayer. The proposed concept of hybrid magnetron sputtering outperformed the commercial PVD coatings of CrN for diesel piston rings manufactured by cathodic arc evaporation.

## 1. Introduction

Ceramic hard coatings are commonly used in high temperature tribological applications due to their high thermal stability, oxidation, and corrosion resistance [1,2]. However, the choice of coating material to be used is critical. Hence, it is necessary to understand the nature of the mechanical, thermal, and corrosive solicitations that the material will be exposed to.

Chromium and chromium-aluminum nitrides (CrN and CrAlN), as monolayers, have shown promising results in increasing the lifetime of different components [3,4]. Such ceramic coating compositions are more stable than those more commonly used such as titanium and titanium-aluminum nitrides (TiN and TiAlN), thus exhibiting higher oxidation resistance [5,6,7,8], which allows them to work at higher temperatures without any detrimental effect to their properties. Therefore, hybrid ceramic coatings manufactured from both ceramic compositions are a natural choice to combine both materials in a multilayer with a superlattice structure, which has shown the potential to further increase the properties of coating materials.

Another critical step in coating selection is the choice of the deposition technique. Nitride-base coatings manufactured by physical vapor deposition (PVD) techniques are commonly found in the literature because of their versatility and the high control of the deposition process [1,9,10]. Among such PVD techniques, magnetron sputtering produces dense and hard ceramic coatings [1,11]. Direct current and high-power impulse magnetron sputtering (dcMS and HiPIMS, respectively) are often used when considering sputtering-based techniques. The former is a well-established technique to provide coatings with good tribological properties and a higher deposition rate. Still, the coating microstructure usually contains voids between the columnar grains, which often limits its hardness and wear performance [12]. Furthermore, the applied power is usually limited by the excessive heating of the targets [12].

HiPIMS, on the other hand, is a relatively novel deposition technique that has been explored and studied over the last couple of decades and consists of a pulsed power technique with a low duty cycle (usually less than 10%), which allows for the use of an elevated peak power while keeping the same average power, thus avoiding overheating issues [13]. HiPIMS produces dense coatings with a few defects and enhanced hardness; however, it is associated with a low deposition rate [14,15].

Some studies have shown that it is possible to combine both techniques and develop a hybrid process that allows for a higher deposition rate (than HiPIMS) and increased hardness (than DC) [16,17]. Furthermore, if different materials are grown on each other, a multilayered architecture will be produced and a superlattice structure may be generated, if the crystal lattices are of the same nature and the periodicity of the sub-layers is small enough.

Although there are plenty of studies on HiPIMS and dcMS producing monolithic coatings and some studies with hybrid magnetron sputtering processes [16,18,19,20], to the best of the authors’ knowledge, there has been no systematic investigation showing how the combination of the target and powering conditions (operating either as HiPIMS or dcMS) affects the mechanical and tribological properties of the coating. Furthermore, it is known that the presence of a base layer for these coatings is responsible for acting as a smooth gradient of properties between the substrate and the coating, thus enhancing the adhesion. Still, it is not clear whether simply applying a nitrided layer on the stainless-steel ring would be enough to promote an adequate gradient and whether a multilayered structure at the base layer would be beneficial.

This study therefore aims at identifying how the combination of the target/power source and different base layer concepts (including their absence) influence the hardness and wear resistance of CrN/CrAlN multilayers.

## 2. Materials and Methods

### 2.1. Coating Deposition

All ceramic multilayer depositions were performed in a Plasma-HiPIMS-250 (PLASMA LIITS, Campinas, Brazil) vacuum chamber equipped with three different target positions and connected to HiPIMS and DC power supplies. The vacuum chamber schematic with three targets used in this work is published elsewhere [17]. The depositions of the coatings were carried out on piston rings made of AISI440 martensitic stainless steel produced for a diesel engine application and supplied by MAHLE Metal Leve, Jundiai, Brazil. The rings had an outer and inner diameter of 131 and 126 mm, respectively, and a thickness of 3.5 mm. The supplier provided the rings in as-nitrided conditions, in which the rings were gas nitrided to produce an approximate 60 μm-thick nitrided layer. The rings were degreased and cleaned with acetone to remove any residual oils from the previous turning steps. Afterward, the substrates were cleaned inside the deposition chamber using Cr-based ion etching (bias set to −700 V on the substrate) to remove all of remaining surface impurities and simultaneously implant a thin layer of Cr ions. The experimental parameters are reported in Table 1. Chromium and chromium-aluminum (50–50 at.%) targets with 99.5% purity and dimensions of 214 mm × 106 mm × 12 mm were used for the coating deposition.

**Figure 1:**
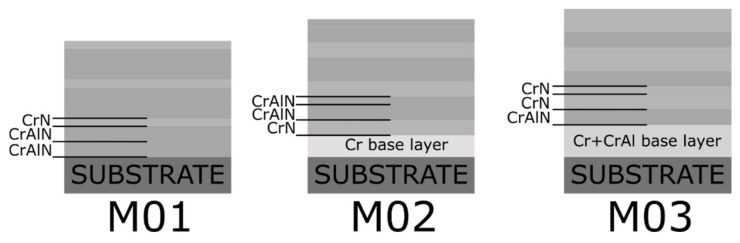
The expected layer distribution based on the different experimental conditions explored in this study including different target combinations and different base layers.

The targets were paired with the different power supplies (either HiPIMS or dcMS) to produce the other experimental conditions explored in this study, see Table 1. Three different experimental conditions for the base layer were investigated: M01 was produced without a base layer, M02 with a metallic Cr layer, and M03 with a Cr and CrAl multilayer. Figure 1 shows the schematics of the layers expected to be obtained considering the different deposition rates for the other targets and power supply units.

The base layers were deposited for 2 h, followed by 20 h of multilayer coating deposition. Based on previous studies [4], the mean power was set to 900 W, a frequency of 500 Hz was used, and a T_on_ of 200 µs, which yielded a duty cycle of 10%.

### 2.2. Coating Characterization

The coating morphology and topography were characterized by a field emission scanning electron microscope (FE-SEM) Inspect F-50 (FEI, Hillsboro, OR, USA). Then, an atomic force microscope Nanosurf Flex (Nanosurf, Liestal, Switzerland) was used to map the topography within an area of 30 μm × 30 μm at the top surface of each coating.

X-ray photoelectron spectroscopy (XPS) was conducted to evaluate the chemical depth profiles through the multilayers using a Versaprobe PHI 5000 (Chanhassen, MN, USA) spectrometer with a 0.2 eV energy resolution, 15 eV Al kα radiation, and a selected area of 200 × 200 µm^2^.

X-ray diffraction was used to determine the phase composition, texture coefficient, and multilayer periodicity with a Rotaflex—RU300B (Rigaku, Tokyo, Japan) diffractometer equipped with a CuKα rotative anode (λ = 1.5406 Å). Data were collected in the range of 35° ≤ 2θ ≤ 85°, with a beam acceleration of 40 kV, a current of 60 mA, a step size of 0.05°, and an acquisition time of 5 s/step. The texture coefficient was calculated by using Equations (1) and (2) below, while the standard intensity of each (hkl) plane was acquired from the ICSD data (card number 00-001-0065).

(1) Thkl=IhklmAθ/2θθhIhklICSD.∑hklIhklICSD∑hIhklmAθ/2θθh

(2) $$A_{\theta /2\theta }\left(\theta_{\mathrm{hkl}}\right)=1-e^{-\frac{2\mu t}{\sin\theta }}$$

The multilayer periodicity was calculated based on Equation (3), using the satellite peaks present in the X-ray diffractograms.

(3) $$\Lambda_{\mathrm{XRD}}=\frac{\left|m-n\right|\lambda }{2\left|\sin\left(\theta_{m}\right)-\sin\left(\theta_{n}\right)\right|}$$

Residual stress analyses were conducted in a MRD-XL PANalytical diffractometer (Malvern, UK) with Mo-Kα radiation to determine the mean (CrN + CrAlN) multilayer stresses using the sin^2^ψ technique applied to the (440) and (424 + 600) diffraction lines of the fcc-CrN phase. Seven ψ-tilts were conducted until the sin^2^ψ-value of 0.9, maintaining an equal spacing in the sin^2^ψ-scale. The Eshelby–Kroener model [21] was employed to calculate the diffraction elastic constants (DEC) using the single crystal elastic constants for CrN [22].

The nanoindentation technique was used to measure the hardness and elastic modulus of the coatings using the Oliver and Pharr method [23] with a PB1000 Mechanical Tester (Nanovea, Irvine, CA, USA) equipped with a Berkovich tip. A total of 15 measurements (a matrix of 5 × 3) were conducted on top of each coating, with a maximum applied load of 30 mN and a load/unload rate of 60 mN/min.

Reciprocating wear tests were performed in the coated rings against a gray cast iron cylinder liner. Four rings of each experimental condition were tested. The testing temperature was set to 130 °C, with an average load of 360 N, at 900 rpm, and a 0W20 oil with Al_2_O_3_ particles (5 g/L) to simulate the engine conditions. The linear wear (both for the ring and cylinder liner) was quantified to compare the different multilayers. A commercial CrN coating deposited by cathodic arc [24] onto the gas-nitrided piston ring was used as a reference of wear resistance.

The mechanical and tribological characterization had two different objectives. While the former intended to assess how the different target/power supply combinations influenced the hardness, the latter aimed to understand how the different base layer concepts affected the adhesion between coating and substrate (i.e., if the coatings were going to detach from the substrate during the wear test).

## 3. Results and Discussion

**Figure 2:**
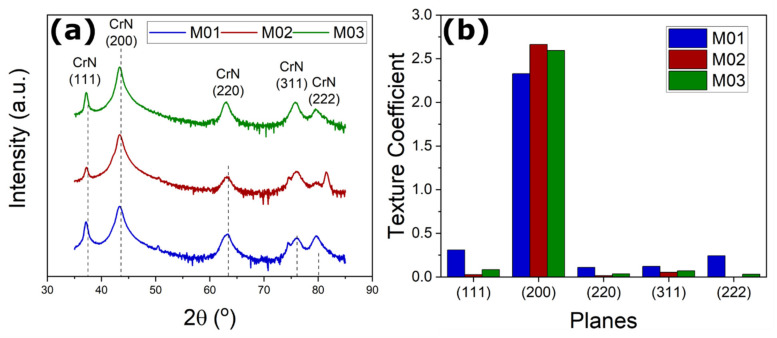
The diffractograms (**a**) of the multilayers deposited under the M01, M02, and M03 experimental conditions showing the presence of CrN peaks and satellite peaks and (**b**) the texture coefficient analysis.

Figure 2a shows the diffractograms of the ceramic multilayers manufactured under the M01, M02, and M03 experimental conditions. In the figure, the dashed vertical lines represent the position and relative intensity of the peaks of the CrN phase. All diffracted peaks corresponded to the face centered cubic (fcc) CrN phase. The absence of peaks related to Cr_2_N or AlN phases showed that aluminum occurs as a substitutional element without phase segregation or AlN precipitation [17,25,26]. In addition, all the peaks presented a slight shift to the left (i.e., toward smaller 2θ angles). This behavior was attributed to the compressive residual stresses on the plane parallel to the surface, which in turn led to a Poisson expansion in the direction of the surface normal [27]. Moreover, Figure 2b displays the texture coefficients for the different lattice planes in the surface normal direction, and a strong (100)-oriented growth was observed for all conditions.

**Figure 3:**
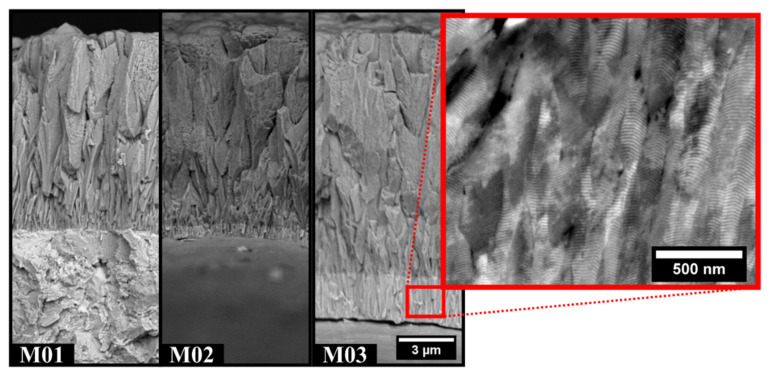
The fractured cross-sections using backscattered signal showing the multilayer thickness and the presence of a thicker base layer for M03. In detail, an electron channeling contrast image shows the multilayer structure present in the base layer.

The FE-SEM micrographs of the fractured coating cross-sections are shown in Figure 3. Columnar growth with a few voids between the grains was found for all multilayers. Such features can be explained by the higher number of dcMS power sources with a higher deposition rate significantly influencing the coating morphology. Another possible mechanism is given by the model proposed by Anders [28]. The formation of well-defined columnar structures with tiny voids in between can be correlated to the low energy during the deposition, which limits the atomic mobility once they reach the substrate surface.

A thicker base layer was observed for the coating manufactured under the M03 experimental condition. In this case, nitrogen (N_2_) was not injected, and all three targets were switched on during the base layer deposition, thus resulting in a multilayered metallic (Cr + CrAl) base layer. Furthermore, the detailed view of the base layer (inset of Figure 3) shows a layered structure of Cr (light contrast) and CrAl (dark contrast), while some of the grains can be observed by electron channeling contrast (ECCI).

The periodicity of the CrN/CrAlN multilayer (20.7 nm) can be measured from the BSE image and matches the prediction given by Equation (4) (20.8 nm) [17].

(4) $$\Lambda \;\left[\mu m\right]=\frac{\mathrm{thickness}\;\left[\mu m\right]}{\mathrm{time}\;\left[h\right]\times \mathrm{speed}\;\left[\mathrm{rpm}\right]\times 60}$$

Measurements of the coating thickness were performed based on the FE-SEM images in Figure 3, and the results are presented in Table 2. The multilayers grown with two CrAl targets (M01 and M02) exhibited a lower thickness than the one deposited with two Cr targets for the same deposition time. This was explained by the lower deposition rate of each nanolayer of CrAlN when compared to CrN.

Table 2 also shows how the periodicity changes for the coatings depending on the target/power source configuration. A comparison between the values predicted by Equation (4) and the value calculated from X-ray diffraction using Equation (3) is provided, and good agreement between the two methods was observed. The only exception occurred for coating M03, in which no satellite peaks were observed. This indicates that in this case, a single layer of the Cr_x_Al_1−x_N compound was formed instead of a multilayered structure.

**Figure 4:**
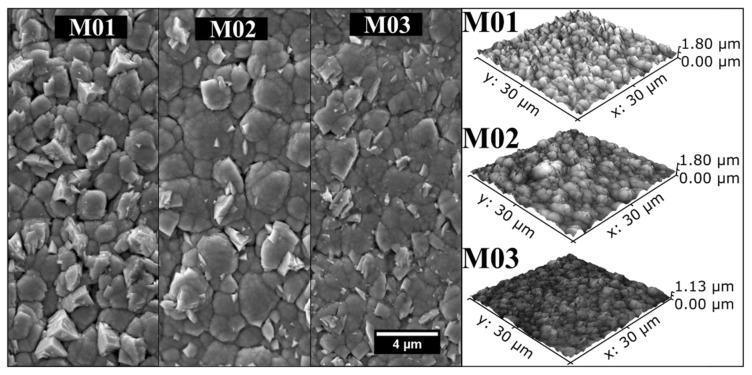
The top surface characterization of the M01, M02, and M03 multilayers by secondary electron FE-SEM imaging and AFM revealed a mix of angular and rounded grains for all deposition conditions.

Figure 4 shows the secondary electron FE-SEM images of the top surface of the coatings, with the AFM maps of the surface topography beside them. The AFM results agreed well with the FE-SEM images. Sharp and rounded grains occurred concomitantly with a few voids between them for all deposition conditions, and no appreciable difference was verified. The mean roughness values, displayed in Table 2, were calculated based on the data collected from the AFM mappings in Figure 4 and indicated slightly higher values for M01 and M02 than M03.

**Figure 5:**
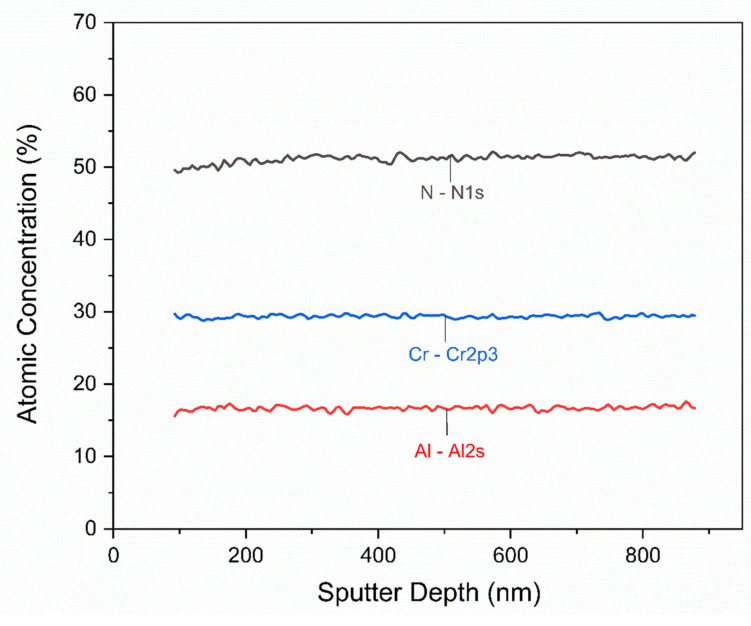
The XPS depth profile of the chemical composition across the CrN/CrAlN multilayer deposited onto a gas-nitrided diesel piston ring using the M02 concept.

The XPS depth profiles were conducted for all the multilayers investigated here, and no significant differences could be observed between the different deposition concepts. Figure 5 exemplifies the XPS depth profile for condition M02. Although the deposition was performed with alternating targets to generate a multilayer architecture, the content of Cr, Al, and N across the multilayer remained invariant. The average Cr, Al, and N content was 29.5, 16.7, and 52 at%, respectively. This reveals that at 400 °C, homogenization occurred due to the formation of wavy interfaces between the CrN/CrAlN sub-layers.

**Figure 6:**
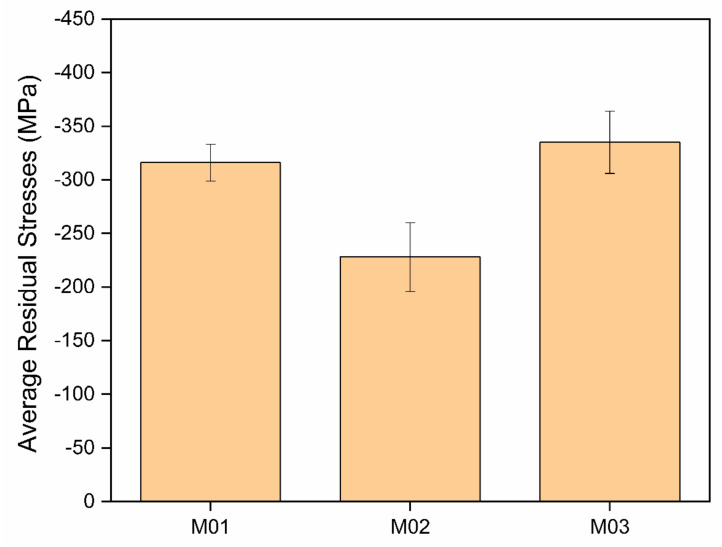
The residual stresses in the CrN/CrAlN multilayers deposited onto the gas-nitrided diesel piston rings as a function of the sputtering conditions.

The mean residual stresses in the multilayers are displayed in Figure 6. In general, the compressive stresses indicated a major influence of the intrinsic stress component caused by the ion bombardment during the physical deposition process. The residual stresses were, however, much smaller than in the CrAlN coatings produced by cathodic arc evaporation [29], due to the slow layer-by-layer atomic removal and consequently lower kinetic energies of the metal ions during the sputtering processes. The M01 and M03 conditions showed the highest compressive stresses. According to the schematic multilayer architectures in Figure 1, this suggests an effect of the base layer on the residual compression in the multilayer. The M02 condition had a metallic Cr base layer, which was softer and seemed to lead to stress relief and accommodation within the multilayer. In both the M01 and M03 conditions, the multilayer grew either on a hard multilayered base layer or directly on the gas-nitrided and hardened piston ring. This caused stress accumulation within the ceramic multilayer.

**Figure 7:**
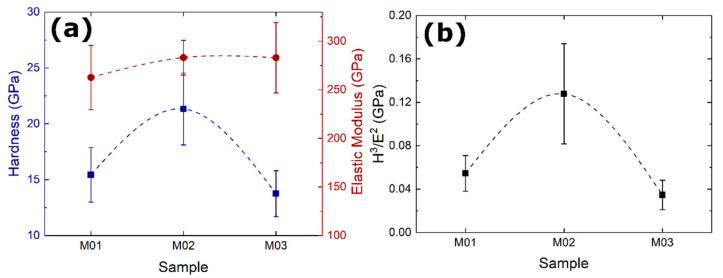
(**a**) The hardness and elastic modulus for each sample showing the maximum hardness for the coating produced with a CrAl target switched to the HiPIMS power supply (M02) and (**b**) calculated H^3^/E^2^ ratio for all three samples, showing the highest ratio for M02.

The effect of the deposition condition on the elastic modulus and hardness of the multilayers was analyzed by instrumented nanoindentation (Figure 7a). The elastic modulus did not undergo an appreciable variation, ranging between 262 and 283 GPa for the different conditions. Meanwhile, the M02 condition produced the maximum hardness of 21 ± 3 GPa, whereas M01 exhibited 15 ± 2 GPa and M03 was softer with 14 ± 2 GPa. Since CrAlN coatings are harder than CrN, M01 and M02 were the hardest coatings due to the increased aluminum content. In addition, HiPIMS was applied to the growth of CrAlN in M02, which further enhanced the hardness of the CrAlN sub-layer in comparison to M01, thus generating the maximum hardness among the deposition conditions investigated.

Based on the hardness measurements, no superlattice hardening effect was observed in the coatings and there are a few reasons why no such effect was observed. As described by [1], superlattice hardening usually occurs when there is either a relevant difference in the shear modulus between the pairing materials or a significant difference in the lattice parameters, and a higher increment in hardness is observed when both are present. When this happens, the superlattice structure provides a substantial barrier for the movement of dislocations through the coating, thus increasing hardness. However, CrAlN and CrN did not exhibit an appreciable difference in neither the shear modulus nor lattice parameter. Hence, what was observed is a rule of mixtures between both phases for M01 and M02. Furthermore, the X-ray analysis of the M03 coating with minimum hardness revealed no satellite peak, thus indicating the absence of a superlattice formation.

The H^3^/E^2^ ratio (Figure 7b), in which H represents the measured hardness and E the measured elastic modulus, is commonly used as a qualitative indicator of the resistance to plastic deformation (i.e., toughness) of the coating material [30,31]. In the present case, the likelihood of plastic deformation in the M02 coating (0.13 ± 0.05 GPa) was hence markedly reduced, followed by M01 (0.05 ± 0.02 GPa) and M03 (0.04 ± 0.01 GPa). When comparing the three coatings, the elastic moduli did not appreciably vary, however, M02 exhibited the highest hardness, which explains the highest toughness for M02. This can be attributed to the positive contribution of the HiPIMS process during the deposition of the hardest CrAlN in the M02 concept.

**Figure 8:**
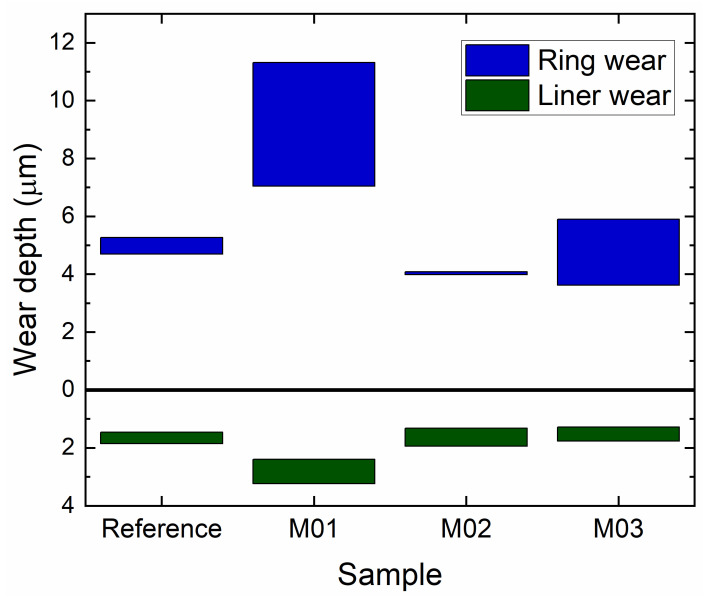
A comparison of the wear depth in the multilayers and coating after the reciprocating wear test, showing a better performance for the M02 deposition condition.

The reciprocating wear test results in Figure 8 showed a higher wear depth for the M01 coating, which had no base layer. The wear depth measured for the M01 coating (9.2 ± 2.1 μm) was approximately equal to the total thickness of the coating (11.4 ± 0.3 μm), which indicates that almost the entire coating delaminated and was removed during the test. The breakage of the coating led to three-body abrasion with fragments of the hard coating acting as abrasive particles in the contact interface, which further increased the wear of the coating and the cylinder liner. This corroborates the importance of the base layer under tribological loadings to smooth the gradient of the mechanical properties across the coating/substrate interface.

The wear resistance of the M02 coating significantly improved (approximately 20% lower wear) in comparison to the state-of-the-art CrN coating produced by arc evaporation, while keeping the same wear depth on the cylinder liner. This behavior was attributed to the use of the HiPIMS process to deposit the harder CrAlN layer, thus producing a denser and more intact microstructure, along with the maximum hardness, when compared to the other deposition conditions in this study, which therefore contributed to the enhanced tribological performance. Furthermore, the highest multilayer toughness, as indicated by the H^3^/E^2^ ratio, obtained with the M02 concept, revealed good agreement with the best tribological performance among the different coatings.

## 4. Conclusions

In the present study, the mechanical and tribological behavior of the CrN/CrAlN multilayers could be tailored using a hybrid magnetron sputtering procedure that combined three targets, two dcMS, and one HiPIMS power supply in three different ways. The relevance of selecting the proper base layer could also be demonstrated through the wear bench tests, which simulated real combustion engine operation conditions, and by comparing the multilayers grown directly onto a gas-nitrided martensitic stainless steel, a Cr/CrAl multilayer, and a pure metallic Cr base layer. The results showed that while the surface roughness and topographic features were invariant to the target/power supply combination, the hardness, toughness, and wear resistance were greatly affected. The ceramic multilayers produced with two targets of CrAl (one switched to HiPIMS and one to dcMS) and one Cr target operating with a dc power supply and grown on a Cr base layer demonstrated the best wear resistance by far among all of the other coatings including the CrN arc evaporated reference coating for the diesel piston rings. This deposition concept yielded good adhesion to the substrate, more elevated hardness, and marked resistance to plastic strain, thus leading to lower wear depths in both the coating and cylinder liner even though no superlattice hardening could be verified. The compressive coating stresses were slightly reduced due to a soft Cr base layer that was strained, enabling stress relief and accommodation within the multilayer. In addition, given the occurrence of a few voids between the columnar grains in all coatings, there could still be room for improvement by experimenting with the increase in the HiPIMS participation in the deposition process and bias conditions to provide more energy to the ionic species, and thus, increase the atomic mobility that may contribute to eliminate voids. Such a strategy could potentially further enhance the mechanical and tribological properties of the multilayers explored here.

## Figures and Tables

**Table 1:** The main deposition parameters for the different coating strategies.

Sample Name		M01	M02	M03
Chamber pressure		2.67 × 10^−1^ Pa		
Bias		−120 V		
Carousel Speed		1.0 rpm		
Base Layer		None	Cr	Cr + CrAl
Base Layer Deposition Time		2 h		
Target (Base layer)		None	Cr HiPIMS	Same as coating
Atmosphere (Base layer)		Ar (40 sccm)		
Atmosphere (Coating)		Ar (40 sccm) and N_2_ (50 sccm)		
Target (Coating)	HiPIMS	Cr	CrAl	Cr
	DC	CrAl	CrAl	Cr
	DC	CrAl	Cr	CrAl

**Table 2:** The deposition rate and periodicity based on the thickness values measured with the SEM images and the roughness calculated from the AFM images.

		M01	M02	M03
**Thickness [μm]**	**Total**	11.4 ± 0.3	11.3 ± 0.1	14.9 ± 0.2
**Deposition Rate [µm/h]**	**Base layer**	-	0.35	1.2
	**Coating**	0.57	0.53	0.63
**Periodicity [nm]**	**Estimated**	9.5	8.7	10.5
	**Calculated by X-ray**	7.1	7.7	-
**Roughness [nm]**	**Ra**	187	183	120

## Data Availability

The original contributions presented in the study are included in the article, further inquiries can be directed to the corresponding author.
